# Aromatase Inhibitors as a Promising Direction for the Search for New Anticancer Drugs

**DOI:** 10.3390/molecules29020346

**Published:** 2024-01-10

**Authors:** Sara Janowska, Serhii Holota, Roman Lesyk, Monika Wujec

**Affiliations:** 1 Department of Pathobiochemistry and Interdisciplinary Applications of Ion Chromatography, Biomedical Sciences, Medical University of Lublin, 1 Chodzki Street, 20-093 Lublin, Poland; 2 Department of Pharmaceutical, Organic and Bioorganic Chemistry, Danylo Halytsky Lviv National Medical University, Pekarska 69, 79010 Lviv, Ukraine; golota_serg@yahoo.com (S.H.); dr_r_lesyk@org.lviv.net (R.L.); 3 Department of Organic Chemistry, Faculty of Pharmacy, Medical University of Lublin, 4A Chodzki Street, 20-093 Lublin, Poland

**Keywords:** aromatase inhibitors, steroidal and non-steroidal compounds, anticancer activity

## Abstract

Aromatase is an enzyme that plays a crucial role in the biosynthesis of estrogens, which are hormones that contribute to the growth of certain types of breast cancer. In particular, aromatase catalyzes the conversion of androgens (male hormones) into estrogens (female hormones) in various tissues, including the adrenal glands, ovaries, and adipose tissue. Given the role of estrogen in promoting the growth of hormone-receptor-positive breast cancers, aromatase has become an important molecular target for the development of anticancer agents. Aromatase inhibitors can be classified into two main groups based on their chemical structure: steroidal and non-steroidal inhibitors. This work presents a review of the literature from the last ten years regarding the search for new aromatase inhibitors. We present the directions of search, taking into account the impact of structure modifications on anticancer activity.

## 1. Introduction

Aromatase inhibitors are a promising direction in the search for new drugs effective in the treatment of cancer, in particular hormone-dependent breast cancer. This is of great clinical importance as breast cancer is the most commonly diagnosed cancer in women, affecting nearly 2.1 million patients each year [1]. Aromatase cytochrome P450 is the only enzyme found in vertebrates that catalyzes the biosynthesis of all estrogens from androgens. Estrogen plays an important role in stimulating the proliferation of cancer cells in patients with estrogen-receptor-positive breast cancer [2]. Therefore, aromatase inhibitors are the first-line therapy for estrogen-dependent breast cancer [3].

Aromatase is a human enzyme belonging to the family of cytochromes P450, constituting the product of expression of the CYP19A1 gene, located on the 15th chromosome [4,5]. Its role in the human body is related to the catalysis of the last stage of estrogen biosynthesis. The last phase of steroidogenesis limits and controls estrogen synthesis in the system. The synthesis catalyzed by aromatase is the aromatization of androgens to estrogens. This chemical transformation requires three oxidation reactions of the A ring of androstenedione, each using oxygen and NADPH. The third stage of oxidation is characteristic of aromatase, while the first two are common to the entire group of cytochrome P450 proteins [6]. The feature that distinguishes aromatase from other cytochrome P450 proteins is its high selectivity toward the substrate. Aromatase as an enzyme is characterized by high androgenic specificity [3]. It has been observed that breast cancer cells show an increased expression of aromatase and, consequently, synthesize higher concentrations of estrogens than normal cells. This relationship is one of the main reasons for the great interest in aromatase as a molecular target for new drugs targeted at cancer pharmacotherapy [7]. 

Since the discovery of aromatase, research into the design of drugs that are aromatase inhibitors has been hampered by the lack of knowledge of its three-dimensional structure. The crystal structure of the human aromatase enzyme was finally solved in 2009 by Ghosh et al. [8,9]. Thanks to the work of this team, we know that the active site of aromatase contains tightly packed hydrophobic side chains that complement the ripples of the steroid backbone, constituting the molecular basis of this enzyme’s high androgenic specificity [9]. Combined with the relatively long path that the steroid has to travel to reach the active site inside the enzyme, this ensures a high level of substrate specificity [7]. Developed by Ghosh et al., the crystal structure of aromatase has enabled [3,8] better drug design based on the structure of the enzyme and also enabled direct analysis of the molecular basis of why some current and past aromatase inhibitors in clinical practice perform better than others [7].

The currently known aromatase inhibitors can be divided into two groups in terms of chemical structure: steroidal and non-steroidal. Steroid inhibitors, also called type I inhibitors, have a spatial structure resembling the native aromatase ligand, which is androstenedione. Due to their similarity to natural aromatase substrates, these inhibitors attach to the substrate binding site of the enzyme. They are then converted to a reactive intermediate that covalently binds to the binding pocket of the enzyme. This leads to the irreversible passivity of aromatase. Type I inhibitors of a steroid structure include the drugs used in clinical practice, such as formestane (withdrawn) and exemestane (still used) [7].

A particularly interesting group from the point of view of organic synthesis and targeted drug design are non-steroidal aromatase inhibitors, also referred to as type II inhibitors. Molecules from this group bind non-covalently to the heme residue found in the aromatase enzyme. The consequence of this is the blocking of the possibility of androgens binding to the molecular target by saturating the site of its binding. Aromatase inhibition by non-steroidal inhibitors, unlike steroidal ones, is reversible due to competitive androgen inhibition [4,9]. Non-steroidal aromatase inhibitors have two basic chemical elements in their structure. One of them is the azole part containing a built-in nitrogen atom that interacts with the iron atom in the heme found in aromatase. The second important element is the extensive aryl part, which makes the molecule similar to the steroid ring characteristic of the natural substrate of the reaction catalyzed by the enzyme [10]. Type II aromatase inhibitors that have been or are used in clinical practice include fadrozol, vorozole, rogletimide, letrozole, and anastrozole [7].

In Drug Bank https://go.drugbank.com/ (accessed on 4 January 2024), there are 30 records of substances with inhibiting activity toward aromatase. Only 5 of them have defined pharmacological activity (aminoglutethimide, anastrozole, exemestane, letrozole, testolactone), for 25, it is marked as unknown, and one has no activity.

**Figure 1:**
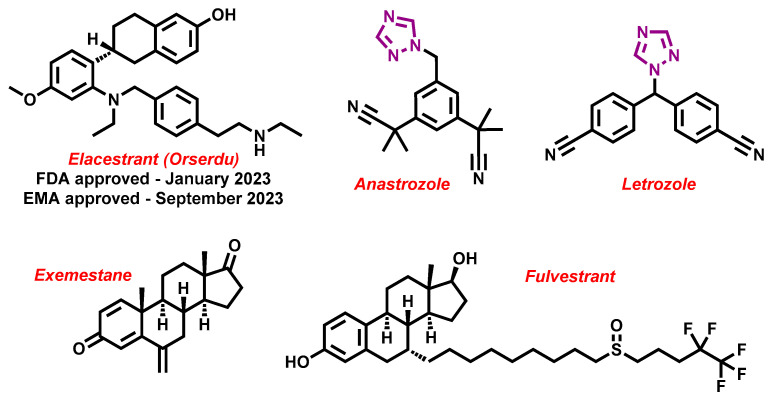
Chemical structures of newly approved aromatase inhibitor elacestrant and early approved aromatase inhibitors used in the combined clinical trials with kinase inhibitors in the period of 2013–2023.

Over the last ten years, 626 clinical trials on aromatase inhibitors have been registered. There are 89 research protocols in the clinical trials database. Most of the conducted studies, as many as 53, concern the possibility of using protein kinase inhibitors and known aromatase inhibitors (Table 1). This mainly concerns preoperative treatment. Three clinical studies demonstrate the possibility of using brilanestrat and amcenestrat—selective estrogen receptor degraders. One of the studies on brilanestrat was suspended due to its toxicity. Eight clinical trials concern the possibility of combined use of monoclonal antibodies and aromatase inhibitors. Two studies concern the simultaneous use of aromatase inhibitors and vitamins B12 and D. Individual studies indicate the possibility of using known drugs used in other types of cancer and also in the treatment of estrogen-dependent breast cancer. Thus, degarelix, elacestrant, and combinations of azacitidine with fulvestrant and enzalutamide with exemetasone are being tested. One study ended with the drug being introduced to the market in 2023. It involved demonstrating the activity of elacestrant (Figure 1) and its effectiveness compared to aromatase inhibitors used in medicine.

In recent years, there have been many papers on the design, synthesis, and testing of the biological activity of new aromatase inhibitors.

## 2. Materials and Methods

**Figure 2:**
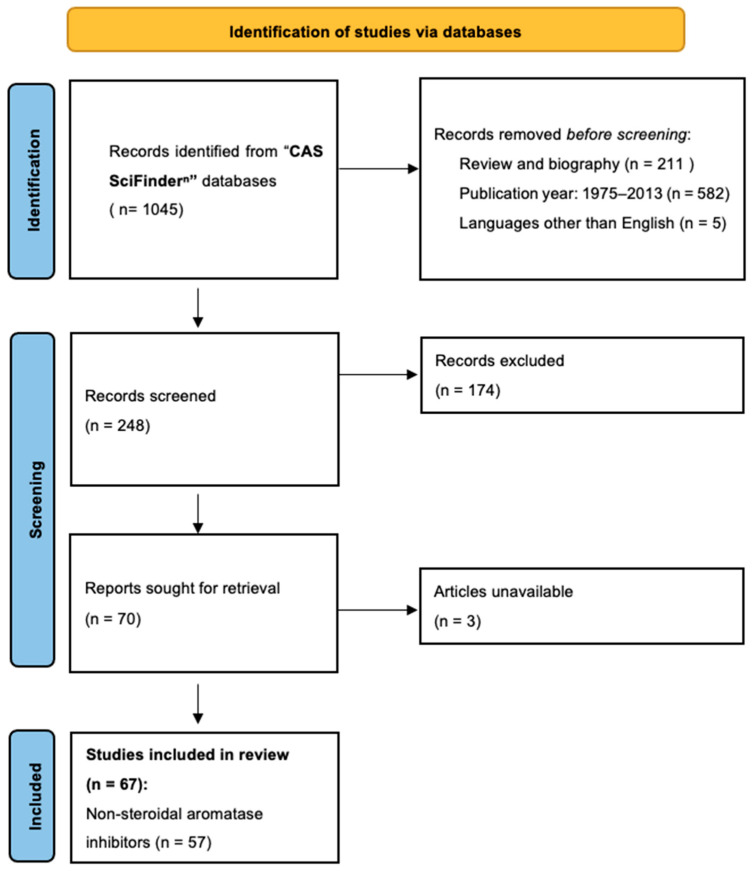
PRISMA flow chart for systematic review [11].

In September 2023, we searched for adequate literature in “CAS SciFinder^n^;” databases using “aromatase inhibitors” AND “synthesis” formulas. The time of publication was limited to years between 2014 and 2023. After searching the literature, data were abstracted, and selected articles were scanned to eliminate studies with irrelevant topics or duplicate records. Reviews were also excluded. Only English language papers were analyzed. We obtained 248 original articles. Ultimately, we selected 67 research papers on the synthesis of potential new aromatase inhibitors and the assessment of their biological activity (Figure 2).

## 3. Aromatase Inhibitors

### 3.1. Steroidal Aromatase Inhibitors

**Figure 3:**
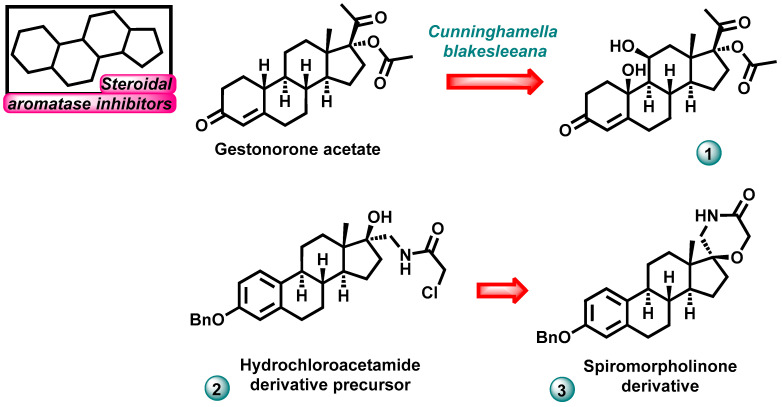
Novel steroidal aromatase inhibitors.

In 2023, Choudhary et al. conducted the synthesis of a new derivative 17α-acetoxy-10β,11β-dihydroxy-progesterone (compound **1**, Figure 3) using the method of biotransformation of gestonorone acetate using the fungus *Cunninghamella blakesleeana* (ATCC 8688). Derivative **1** was described as a non-cytotoxic inhibitor of the human aromatase enzyme with an IC_50_ value of 0.827 µM. Exemestane was used as the reference drug, and its aromatase activity was estimated at IC_50_ = 0.232 μM [12].

In the same year, Cobos-Ontiveros et al. published the results of research on the development of a series of aromatase inhibitors with a steroid skeleton and a heterocyclic system in their structure. The obtained compounds were assessed for their antiproliferative properties in relation to estrogen-dependent and estrogen-independent cell lines: lung cancer cell A549, cervical cancer cell line HeLa, breast epithelial cell line HBL-100, lung cancer cell line SW1573, human breast cancer cell line T-47D, and human colon carcinoma cell line WiDr. Two derivatives (compounds **2**, **3**, Figure 3) from the newly synthesized series showed strong anticancer activity against six cancer cell lines. In the tests, the most active compound had GI_50_ values in the range of 0.25–2.4 μM. Abiraterone (GI_50_ values in the range of 24–100 μM) and galeterone (GI_50_ values in the range of 2.1–10 μM) were used as reference drugs. To explain the mechanism of action of the compounds, molecular docking tests were performed, which demonstrated the ability of active molecules to inhibit aromatase [13].

**Figure 4:**
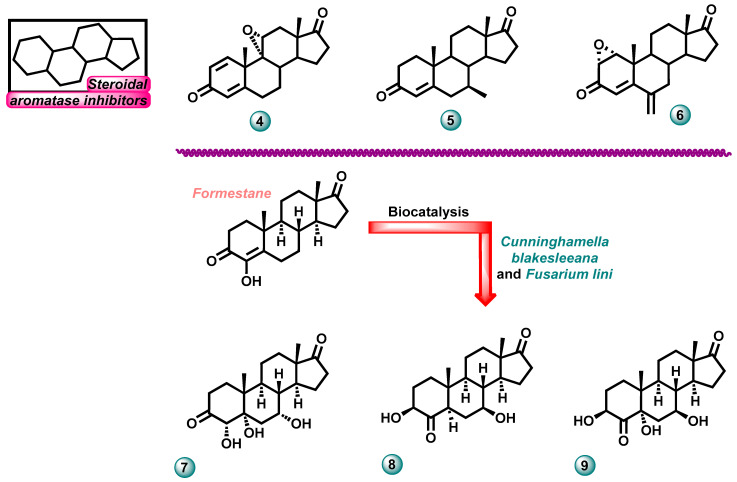
Examples of new potential aromatase inhibitors with steroidal scaffolds.

In 2023, Roleira et al. carried out work as a result of which a series of steroid molecules that were potential aromatase inhibitors were designed, synthesized, and tested for biological activity. In one series, steroids substituted at the C-11 position with an α or β hydroxyl group or a carbonyl group were tested, as well as C-9/C-11 olefins and steroid epoxides. It was found that for aromatase inhibitory activity, it is advantageous to place a carbonyl group at C-11 in the molecule. In this way, a very strong aromatase inhibitor was obtained—compound **4** (Figure 4)—with an IC_50_ against this enzyme of 11 nM. Its activity was higher than the reference exemestane (IC_50_ = 50 nM). This subsequently led to the discovery of the most potent steroid aromatase inhibitor in the published study—molecule **5** (Figure 4)—with an IC_50_ of 5.8 nM. Compound **5** showed more favorable aromatase activity than compounds currently used in clinical practice [14].

Amaral et al. conducted research aimed at examining the anticancer properties and multidirectional mechanism of action of the compound 1α,2α-epoxy-6-methyleneandrost-4-ene-3,17-dione (oxy) (compound **6**, Figure 4). This molecule is a derivative of exemestane. To determine its activity, the effect of the newly synthesized compound was tested on estrogen-receptor-positive (ER+) breast cancer, which is characterized by aromatase overexpression on MCF-7 cells, as well as on the breast-cancer-cell-line-resistant LTED. The tests showed that derivative **6** reduced the expression and activation of ERα and induced the overexpression of aromatase with an effect promoting cell death. Complementary transactivation assays showed that molecule **6** exhibited ER antagonist and AR agonist activity [15].

New derivatives of the anticancer drug formestane were developed by Wahab et al. (compounds **7**–**9**, Figure 4). These compounds were synthesized through biocatalyzed structural modifications carried out by *C. blakesleeana* and *Fusarium lini*. The new derivatives showed different abilities to inhibit the aromatase enzyme. The most active compound **9** showed significant inhibitory potential with an IC_50_ of 0.386 μM, comparable to the parent drug for which the IC_50_ value was 0.335 μM [16].

**Figure 5:**
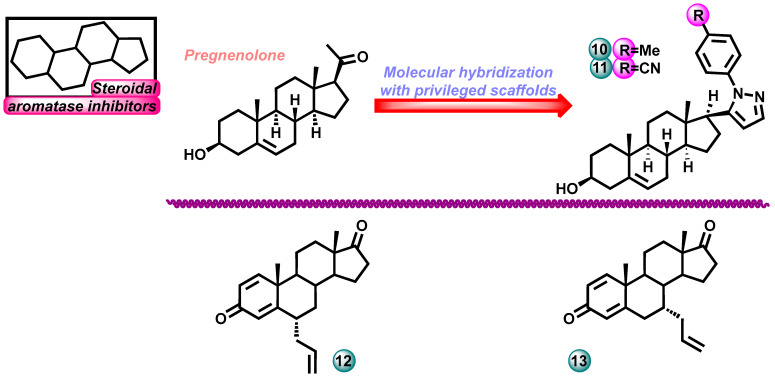
Novel steroidal aromatase inhibitors.

In the same year, Banday et al. conducted research on the synthesis and assessment of the biological activity of two classes of structurally and functionally diverse pregnenolone pyrazoles (compounds **10**, **11**, Figure 5). These compounds were designed as aromatase inhibitors that are antiproliferative agents. New steroid derivatives were tested for their aromatase enzyme inhibitory activity using a dibenzylfluorescein (DBF)-based fluorescent assay. The study demonstrated the significant potential of pregnenolone pyrazoles **10**, **11** as aromatase inhibitors with potential use in the treatment of breast cancer. Molecular-docking-based studies have demonstrated effective binding of new steroid analogs to the human aromatase enzyme [17].

The design and synthesis of a series of new steroidal androstanes with various substituents at the C-6 and C-7 positions were presented in 2019 by Roleir and colleagues. Among the derivatives obtained by the team, compound **12** (Figure 5) stood out with its ability to inhibit aromatase, with IC_50_ values of 55 nM and Ki = 22.5 nM (irreversible inhibition). The reference drug used in the study was formestane with an IC_50_ value of 42 nM. The study concluded that the C-6α position is more favorable than the C-7α position in terms of the compound’s activity toward the aromatase enzyme. Moreover, the substituents that had the most beneficial effect on the inhibition ability were the methyl group, the allyl group, and then the hydroxyl group [18].

Amaral et al. in 2017 published a paper in which they presented research on the biological activity of a series of 7α-substituted steroid molecules. The study examined their aromatase inhibition ability and anticancer potential against sensitive and resistant breast cancer cell lines in vitro. All new steroids effectively inhibited aromatase in tests. The most active compound **13** (Figure 5) had an IC_50_ of 0.3 µM against MCF-7. The reference drugs in the study were formestane and exemestane with IC_50_ values of 0.112 and 0.9 µM [19].

**Figure 6:**
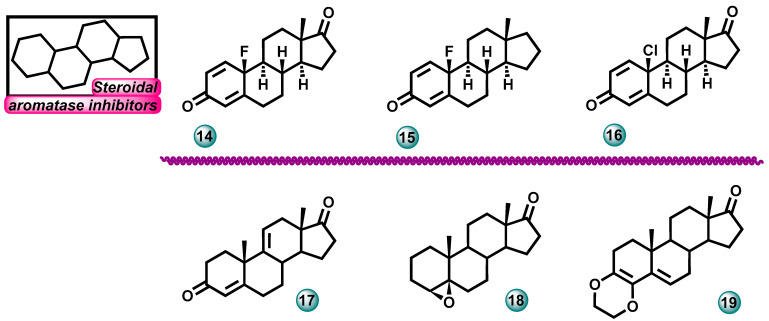
New steroidal molecules as possible aromatase inhibitors.

A study on the aromatase inhibition ability of 10-fluoro- or 10-chloroestra-1,4-dien-3-one derivatives (compounds **14**–**16**, Figure 6) was conducted by Jójárt et al. The compounds were obtained by the fluorination of the hormonally inactive 13α-estrone. The obtained molecules were tested in an in vitro test for their ability to inhibit the aromatase enzyme. The tested compounds containing the 13β-Me group showed strong inhibitory effects with submicromolar and micromolar IC_50_ values of 0.49, 5.0, and 2.4 µM. The reference compound in the study was androst-4-ene-3,17-dione with an IC_50_ value of 0.22 µM [20].

Varela et al. in 2016 carried out research on the synthesis of new steroids containing heterocyclic dioxene condensed in the A ring (compounds **17**–**19**, Figure 6). Biological activity tests and studies were also carried out to explain the mechanisms of action of the obtained molecules. The most active compounds had IC_50_ values for aromatase inhibition in the range of 0.11 (for compound **18**)—0.25 µM (for compound **17**). Some 5β-steroids showed aromatase-inhibiting activity in in vitro tests because they adopt a similar A-ring conformation to androstenedione, a natural aromatase substrate [21].

### 3.2. Non-Steroidal Aromatase Inhibitors

#### 3.2.1. Azoles

##### Imidazoles

**Figure 7:**
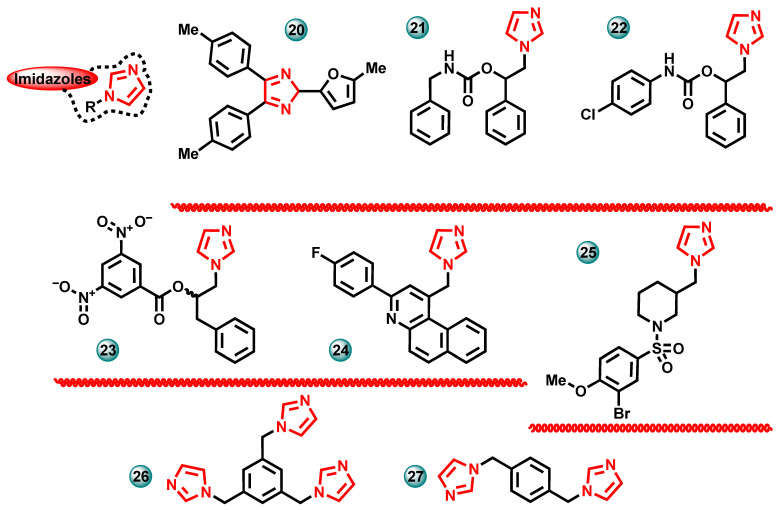
Potential aromatase inhibitors with imidazole scaffolds.

Çetiner et al. (2023) synthesized a series of imidazole derivatives and then evaluated them in vitro for anticancer activity against the MCF-7 human breast cancer cell line in the MTT test. To determine the selectivity of the newly synthesized compounds, their effect on the L929 mouse normal fibroblast cell line was also tested. Three compounds from the synthesized series turned out to be more active against MCF-7 than the used reference drug, which was cisplatin (IC_50_ = 9.75 µM). Compound **20** (Figure 7) with the strongest activity against MCF-7 showed activity with an IC_50_ of 7.9 µM. Imidazole derivatives turned out to be highly selective, showing a lower cytotoxic effect on normal cells than on cancer cells. The compounds were also tested against the aromatase enzyme, in which it was shown that they have lower inhibitory effects on this protein compared to the reference drug letrozole, with an IC_50_ in the range of 5.42–8.79 µM [2].

New carbamate derivatives based on triazole and imidazole were synthesized by Ammazzalorso et al. The obtained compounds were evaluated in an in vitro test against the human aromatase enzyme using the fluorimetric method. Letrozole was used as the reference drug. Afterward, the effect of the most active derivatives on the MCF-7 human breast cancer cell line was assessed using the MTT assay. Measurements using a cytotoxicity assay (LDH release) and cell cycle analysis were also performed. The most active compounds **21** and **22** (Figure 7) showed cytotoxic activity toward MCF-7 with IC_50_ of 6.58 µM and 7.16 µM, respectively. Imidazole derivatives induced a significant inhibition of the aromatase enzyme, with percentages of 81 and 72%, respectively, compared to the reference drug letrozole (100%). The aromatase inhibition activity in the enzymatic test for compounds **21** and **22** was 0.82 µM and 0.86 µM, respectively [22].

In the same year, Maccallini et al. published research results on the synthesis of new imidazole and triazole derivatives [23]. The biological activity of the synthesized compounds was assessed in in vitro tests. They showed that the molecules have a dual mechanism of anticancer activity: the ability to inhibit aromatase and inducible nitric oxide synthase. The most active imidazole derivatives induced a significant inhibition of the aromatase enzyme, with a percentage of 82%, compared to the reference drug letrozole (100%). Additionally, compounds selected from the series were tested for cytotoxic activity against the MCF-7 breast cancer cell line. The most active compound **23** (Figure 7) in the series had an IC_50_ value of 120 μM against MCF-7 [23]. 

The design and synthesis of new imidazole/quinoline derivatives were published by Ghodsi et al. in 2015. The obtained compounds were assessed for cytotoxicity against human breast cancer cell lines MCF-7 and T47D, using doxorubicin as a reference drug (IC_50_ = 0.25 and 0.1 μM). All tested molecules were more cytotoxic to MCF-7 cells (IC_50_ < 5 μM) than to T47D cells (IC_50_ < 5 μM). The results of the team’s work showed that compound **24** (Figure 7) has a higher potency in inhibiting the aromatase enzyme than the reference drug letrozole at a concentration of 5 μM which was used as a positive control [24].

The synthesis of new sulfonamide derivatives of imidazolylmethylpiperidine was described by Di Matteo et al. These compounds were designed as potential aromatase inhibitors. The three newly synthesized compounds showed activity similar to the reference drug letrozole (IC_50_ = 4 nM) in an in vitro aromatase inhibition test. The most active compound **25** (Figure 7) had a potency expressed in an IC_50_ of 6 nM [25].

Kalalinia et al. performed molecular docking simulations in which they showed that four new azole derivatives can effectively interact with the active sites of the aromatase enzyme. The compounds were synthesized and then subjected to in vitro inhibition tests for this protein. The conducted research showed that compounds **26** and **27** (Figure 7) are effective aromatase inhibitors with IC_50_ values of 0.2 nM and 6.8 nM, respectively. These results were similar to the activity of the reference drug letrozole (IC_50_ = 0.3 nM) [26].

##### Benzimidazoles

**Figure 8:**
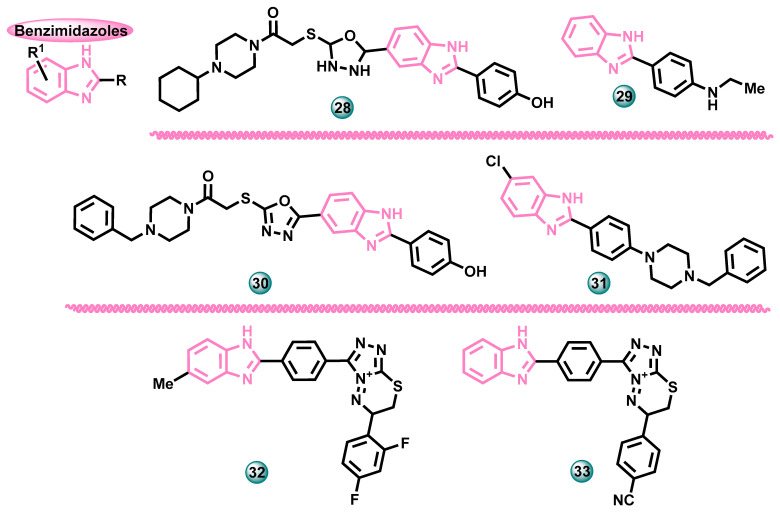
Potential aromatase inhibitors with benzimidazole scaffolds.

In 2023, Acar Çevik et al. conducted research to determine the aromatase inhibition capacity of a series of benzimidazole-bearing hybrids. In the in vitro assay, the antitumor activity of all compounds was tested using the MTT assay against five tumor cell lines (human breast cancer cell line MCF-7, lung cancer cell A549, cervical cancer cell line HeLa, rat glioma cell line C6, and human liver cancer cell line HepG2). Compound **28** (Figure 8) showed high cytotoxic activity against MCF-7 with IC50 = 5.165 µM and HepG2 with IC_50_ = 5.995 µM. In addition, molecule **28** was found to be more active than the reference drug doxorubicin against the HeLa cell line. The selectivity of the cytotoxic effect was assessed by performing tests against the embryonic fibroblast murine cell line NIH 3T3. The tested molecules showed high selectivity. In addition, enzymatic inhibition tests against the aromatase enzyme were performed in vitro. Compound **28** caused inhibition on the aromatase enzyme at 2.314 µM, while reference drug letrozole showed inhibition at 32 nM. A molecular docking simulation was also performed for derivative **28** to determine possible protein–ligand interactions and complex stability. In silico studies performed for the most active compound showed a binding mode comparable to letrozole, and Pi-Pi stacked interaction with residual Phe221 continued during 100 ns simulation, while the molecule remained stable at the active site [27].

In the same year (2023), Sayyad and co-authors designed new derivatives of *N*-(4-(1*H*-benzo[*d*]imidazol-2-yl)phenyl)arylamine and subjected the resulting structures to in silico screening, such as ADMET analysis and molecular docking tests. Selected, most promising molecules were synthesized. The cytotoxic activity of the newly synthesized compounds was assessed using the MTT test against the human breast adenocarcinoma cell line MDA-MB-231, breast cancer cell line MCF-7, lung cancer cell line A549, human lung cell line NCI-H23, and cellosaurus cell line A-498. Doxorubicin hydrochloride was used as the reference drug. Compound **29** (Figure 8) proved to be the most active in the series showing −9.5 kcal/mol binding affinity with molecular target. In vitro, it showed promising activity with GI_50_ values of 0.796 µM, 0.695 µM, 1.14 µM, 2.15 µM, and 0.987 µM toward MDA-MB-231, MCF-7, A-549, NCI-H23, and A-498, respectively [28].

New 1,3,4-oxadiazole-benzimidazole derivatives were designed and synthesized by Çevik at al. The tests then assessed the cytotoxic effects of the newly synthesized compounds against five cancer cell lines, including lung cancer cell A549, cervical cancer cell line HeLa, rat glioma cell line C6, and human liver cancer cell line HepG2, using the MTT assay method. The two obtained compounds showed promising cytotoxic activity against cancer cells, compared to the reference drug doxorubicin (IC_50_ = 10.53 μM). The most active compound **30** (Figure 8) showed anticancer activity against the estrogen-dependent breast cancer cell line with an IC_50_ of 5.13 μM. In vitro aromatase enzyme inhibition tests were also performed to confirm the ability of active molecules to inhibit this protein with an IC_50_ of 1.475 μM (the reference drug was letrozole with IC_50_ = 0.032 μM). Dynamics simulations and bond free energy analyses were also used to better understand the mechanism of action of new aromatase inhibitors, with MMPBSA binding free energy −176.875 kJ/mol [29].

New benzimidazole derivatives with potential anticancer activity were obtained by Saglik et al. The biological activity of the newly obtained compounds was assessed against human cell lines A549 and MCF-7 using the MTT assay. 4-Benzylpiperidine-containing hybrids were found the most active against the MCF-7 cell line, and derivative **31** (Figure 8) with an IC_50_ value of 24 nM was close to the potency of the reference drug cisplatin (IC_50_ = 21 nM). These compounds were then subjected to in silico aromatase enzyme inhibition studies to determine possible protein binding modes and interactions underlying their activity. It has been found that these compounds can be settled very properly to the active site of the aromatase [30].

Benzimidazole hybrids with condensed triazole-thiadiazine rings were synthesized by Cevik et al. The biological activity of the newly obtained compounds was assessed using the MTT test to demonstrate anticancer activity against the estrogen-dependent breast cancer cell line MCF-7 while using cisplatin as the reference drug (IC_50_ = 19 nM). The most active compound **32** (Figure 8) in this test had an IC_50_ of 12 nM. This compound also showed a significant ability to inhibit the aromatase enzyme in in vitro tests with an IC_50_ value of 37 nM (the reference drug was letrozole with IC_50_ = 24 nM). During the research, cytotoxicity tests were also performed against a normal, healthy cell line (NIH3T3), which demonstrated the selectivity of the newly synthesized molecules. Moreover, the interactions between the most cytotoxic compounds and the active site of the enzyme were analyzed by a docking study [31].

The same authors in 2020 published research results on the synthesis and biological activity of sixteen similar compounds—benzimidazole—triazothiadiazine hybrids as potential aromatase inhibitors. The newly synthesized compounds were tested for their cytotoxic properties against the human breast cancer cell line (MCF-7). The most active compounds were further tested for the inhibition of the aromatase enzyme in vitro. This made it possible to determine possible mechanisms of action underlying the activity of the molecules. The most active compound **33** (Figure 8) showed a slightly weaker aromatase inhibitory effect (IC_50_ = 32 nM) than the reference drug letrozole (IC_50_ = 24 nM). This compound also had the highest cytotoxic activity against MCF-7 with an IC_50_ value of 16 nM (the reference drug was cisplatin with IC_50_ = 20 nM) [32].

##### Triazoles

**Figure 9:**
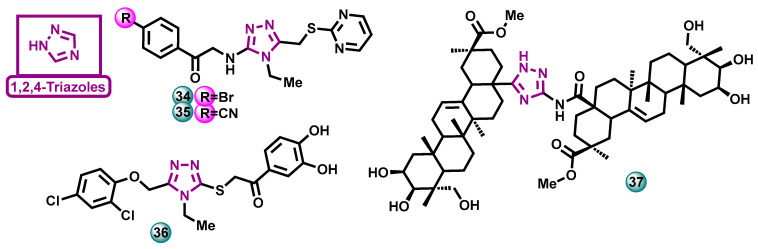
Potential aromatase inhibitors with triazole scaffolds.

Osmaniye et al. [33] synthesized 10 new pyrimidine-triazole derivatives and tested their anticancer activity in vitro. Two compounds, designated as **34** and **35** (Figure 9), showed the most promising activity against the MCF-7 cell line with IC_50_ values of 1.573 and 3.698 μM, respectively. For the reference drug doxorubicin, the IC_50_ value was 0.958 μM. They also showed the ability to inhibit aromatase in in vitro tests, with IC_50_ = 0.082 µM for **34** and 0.198 µM for **35** (the reference drug was letrozole with IC_50_ = 0.031 μM) [34].

The same authors in 2021 conducted research aimed at developing new aromatase inhibitors containing a 1,2,4-triazole ring in their structure too. During the work, a series of compounds were synthesized, and then a cytotoxicity test (MTT) was performed on them to determine their anticancer activity against the estrogen-dependent breast cancer cell line (MCF-7). In this test, the most active compound **36** (Figure 9) had anticancer activity with an IC_50_ of 7.501 µM against MCF-7 (the reference drug was doxorubicin with IC_50_ = 16.385 µM). To determine the selectivity of their action, the obtained triazole derivatives were screened against the healthy NIH3T3 cell line (mouse embryonic fibroblasts). The activity against normal cells was lower than against cancer cells. In vitro studies established that the most active compound **36** had an inhibitory potential of the aromatase enzyme with an IC_50_ value of 0.028 µM (the reference drug was letrozole with IC_50_ = 24 nM) [34].

Xi et al. in 2020 developed compound **37** (Figure 9) which inhibits the viability of ovarian cancer cells and directs them to the path of apoptosis. The aim of the research was to find molecular targets for this molecule and to assess its intracellular signaling in ovarian cancer cells. Ultimately, it turned out that this compound does not work by inhibiting aromatase but by reducing the expression of CYP19A1 mRNA responsible for aromatase synthesis [35].

**Figure 10:**
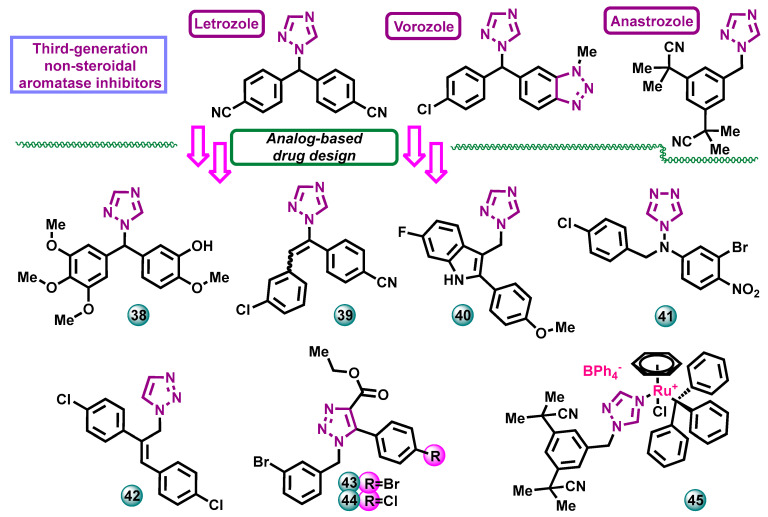
Potential aromatase inhibitors with triazole scaffolds as analogs of approved drugs.

A paper on the synthesis and assessment of the biological activity of a series of heterocyclic diaryl-methanes was published by Ana et al. The molecules were designed as potential aromatase inhibitors. An in vitro study on the MCF-7 breast cancer cell line showed that the 1,2,4-triazole derivative **38** (Figure 10) had the highest activity among the synthesized series. The IC_50_ value for hit compound **38** of 52 nM was compared with the reference drug Combretastatin A4—3.9 nM [36].

Vosooghi et al. in 2014 published results on the synthesis of a series of compounds, in the reaction route starting from 4-bromotolunitrile, which was reacted with 1*H*-1,2,4-triazole, thereby obtaining 4-(4-cyanobenzyl)-1,2,4-triazole. Further reaction with various aromatic aldehydes led to the formation of a series of designed compounds. Eleven derivatives obtained were tested for their anticancer activity against three human breast cancer cell lines: MCF-7, MDA-MB-231, and T47D. The reference drug used was etoposide (IC_50_ = 13.42 μM). The strongest potential aromatase inhibitor and the most active against the MCF-7 cell line was found to be compound **39** (Figure 10) with an IC_50_ value of 88.36 μM [37].

Hybrid molecules containing 1,2,4-triazole/imidazole and 2-phenylindole moieties were described by Kang et al. The aromatase inhibition potential of compounds was explored in vitro, employing letrozole as a referential drug (IC_50_ = 49.5 nM). The highest inhibitory activity of this protein was demonstrated by compound **40** (Figure 10), having a methoxy group at the aromatic ring, with an IC_50_ value of 14.1 nM. The research was extended to include in silico studies in which the methods of binding letrozole and compound **40** with aromatase were identified [38].

A series of 13 derivatives of 1,2,4-triazole derivatives substituted with the 4-*N*-nitrophenyl fragment was synthesized by Song et al. The ability of the synthesized compounds to inhibit aromatase in vitro was assessed, and the team’s findings suggest that the bioactivity of the derivatives could be augmented by attaching substituents to the benzyl group. The most active compound **41** (Figure 10) had a chlorine substituent on the aromatic ring. It showed an IC_50_ of 9.02 nM in the aromatase inhibition assay, compared to the reference drug, which was letrozole with an IC_50_ value of 2.18 nM [39].

Nielsen et al. in 2019 developed a new method of synthesizing methylstilbene derivatives with a triazole ring as substituent and then tested the inhibitory activity of selected derivatives against human aromatase in vitro. The assay showed that all new molecules in the series inhibited human recombinant aromatase in the low nanomolar range, with a Ki value of 8 nM for the most active compound **42** (Figure 10), utilizing ketoconazole as a positive control. It was observed that halogenated derivatives were more potent inhibitors than their non-halogenated counterparts [40].

A series of compounds containing the 1,2,3-triazole moiety were synthesized by McNulty et al. Some of the obtained compounds showed high potency in inhibiting human aromatase (CYP 450 19A1). The most potent aromatase inhibitors in the series were compounds containing halogen-aryl moieties in positions that mimic the carbonyl substituents of natural enzymatic substrates. The Ki values for the most active compounds **43** and **44** (Figure 10) were 20 nM and 30 nM, respectively [41].

Golbaghi and co-workers obtained complexes of ruthenium η 6-benzene and anastrozole derivatives. These compounds were prepared as anticancer drugs with a mechanism of action based on aromatase inhibition. Of these complexes, one was found to be the most stable in the cell culture medium, leading to the highest cellular uptake (compound **45**, Figure 10). This relationship translates into the highest cytotoxic activity in vitro against two human lines of estrogen-dependent breast cancer, MCF-7 (IC_50_ ≥ 4 µM) and T47D (IC_50_ ≥ 4 µM), using cisplatin as the reference drug (IC_50_ = 37.0 µM against MCF-7 and IC_50_ > 150 µM against T47D). Exposure of zebrafish embryos to compound **45** (12.5 µM) did not lead to any noticeable signs of toxicity over the 96 h test period, making it a promising candidate for further animal testing [42].

#### 3.2.2. Coumarin-Based and Coumarin/Chromone/Heterocycle-Bearing Hybrid Molecules

**Figure 11:**
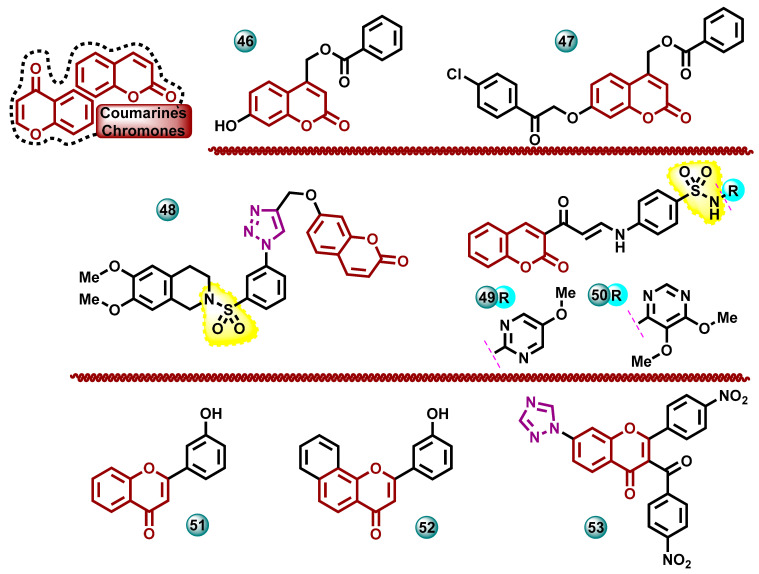
Coumarin-based and coumarin/chromone/heterocycle-bearing hybrid molecules as potential aromatase inhibitors.

A new series of 7-substituted coumarin scaffolds containing a methyl ester moiety at the C4 position were synthesized by Takla et al. in 2023. The obtained derivatives were tested for anticancer activity in vitro against estrogen-dependent breast cancer cell lines MCF-7 and estrogen-independent MDA-MB-231. Doxorubicin was used as the reference drug in the test. Compounds **46** and **47** (Figure 11) showed high selective activity against MCF-7 with IC_50_ values 6.0 and 5.8 µM, respectively, close to the doxorubicin DOX activity of IC_50_ = 5.6 µM. The most promising compounds for activity against MCF-7 were assessed for their ability to inhibit aromatase using exemestane as a reference drug. The results showed that compound **47** had the highest aromatase inhibitory activity with an IC_50_ value of 0.114 µM (94.73% exemestane potency). Compound **46** showed half the aromatase inhibitory activity compared to exemestane [43].

In 2015, Pingaew and co-workers synthesized a series of hybrid molecules containing a coumarin ring linked with 1,4-disubstituted-1,2,3-triazoles containing a sulfonamide moiety. The ability of the synthesized compounds to inhibit aromatase in vitro was tested, using letrozole as the reference drug (IC_50_ = 3.3 nM). Most open-chain sulfonamide triazoles showed significant aromatase inhibition potency, with IC_50_ values ranging from 1.3 to 9.4 µM. Notably, compound **48** (Figure 11) showed the highest activity in the test with an IC_50_ value of 0.2 µM [44].

New chromene-heterocycle hybrids containing sulfonamide moiety and in vitro anticancer activity of these molecules were described by Ghorab et al. The breast cancer cell line T47D was used for investigations. Most of the synthesized derivatives showed high to moderate activity. The most active compound **49** (Figure 11) had an IC_50_ of 8.8 µM and at the same time was more active than the reference drug doxorubicin (IC_50_ = 9.8 µM). In order to determine the mechanism of anticancer activity, the influence of the most cytotoxic compounds on the aromatase enzyme was examined in vitro. Most of the selected compounds showed high aromatase inhibition ability, with the most active compound **50** (Figure 10) having an IC_50_ of 4.66 µM in the test (referent letrozole IC_50_ = 29.5 μM) [45].

In 2022, Sahah et al. carried out work that examined and documented the aromatase inhibitory activity of many natural and synthetic flavonoids. These compounds were tested for their anticancer effects, which could be used in the treatment of breast cancer. The biological potential of 18 compounds selected based on the docking result was assessed. The antitumor activity was tested in vitro against the human breast cancer cell line MCF-7. Of the 18 compounds tested, 7 showed high cytotoxic potential. The most active drug showed activity in tests with an IC_50_ of 0.345 µM, significantly higher than the reference drug, letrozole with an IC_50_ value of 0.86 μM. The ability to inhibit the aromatase enzyme was also assessed using a fluorogenic assay kit. Protein inhibition IC_50_ values for compounds **51** and **52** (Figure 11) were found to be 0.31 µM and 0.36 µM, respectively [46].

The series of new flavone hybrids containing imidazole/triazole moieties were obtained by Sable et al. The synthesized compound **53** (Figure 11) was subjected to biological activity tests regarding its ability to inhibit aromatase in vitro. The molecules’ activity against the MCF-7 breast cancer cell line was also tested using the SRB assay. It was found that the triazole derivative **53** with a nitro substituent on the phenyl groups has an activity close to that of the reference drug letrozole. The IC_50_ for compound **53** was 11.23 µM, while for the reference drug, it was 10.62 µM [47].

#### 3.2.3. Other Heterocycles

**Figure 12:**
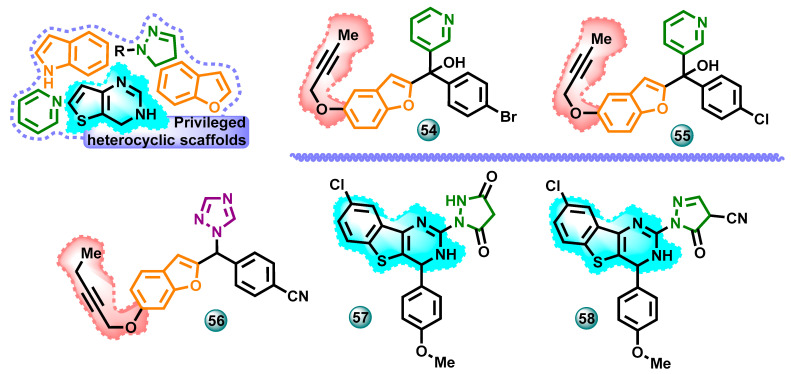
Potential aromatase inhibitors with benzofuran and benzothienopyrimidine scaffolds.

In 2023, Eissa et al. published a synthesis of non-steroidal aromatase inhibitor molecules from the group of benzofuran derivatives. The work was also extended to computational research. Molecule cytotoxicity tests showed that hybrids **54** and **55** (Figure 12) were the most prominent aromatase inhibitors with IC_50_ values of 0.83 nM and 0.92 nM, respectively (cf. letrozole IC_50_ 0.70 nM). Computational studies of pyridine derivatives identified an alternative access channel lined by Phe221, Gln225, Trp224, and Leu477. This allows further insight into the interactions between non-steroidal aromatase inhibitors and their molecular target [48].

New benzofurane-1,2,4-triazole hybrids were developed by Eissa et al. in 2022. To achieve high inhibitory activity against aromatase, the chemical structure previously developed by the team was modified and tested using the available crystal structure of aromatase CYP19A1 (PDB 3S79). The modifications carried out concerned the addition of long-chain substituents, e.g., but-2-ynyloxy and pent-2-ynyloxy, at different positions. In this way, a series of triazole derivatives containing aryl groups in the structure was obtained. For the most active compound **56** (Figure 12), the IC_50_ value was 0.09 nM against aromatase (the reference drug was letrozole with IC_50_ = 0.70 μM) [49].

Sobh and co-workers designed, synthesized, and evaluated the activity of a series of new cytotoxic agents based on a benzothienopyrimidine scaffold. The antitumor activity of these compounds was evaluated against the estrogen-dependent breast cancer cell line MCF-7. The tests used erlotinib and letrozole as reference drugs. As many as eight newly synthesized compounds showed higher anticancer activity than erlotinib in the MTT test, and five of them showed stronger activity than letrozole (IC_50_ = 9.56 µM). In tests against MCF-7 cells, the highest activity was shown by compound **57** (Figure 12) with IC_50_ = 0.87 µM. The most promising compounds were evaluated for their activity against the EGFR (epidermal growth factor receptor) and aromatase enzymes. Compound **58** (Figure 12) with an IC_50_ of 0.146 µM showed the highest aromatase inhibitory activity (letrozole IC_50_ = 43 nM) [50].

**Figure 13:**
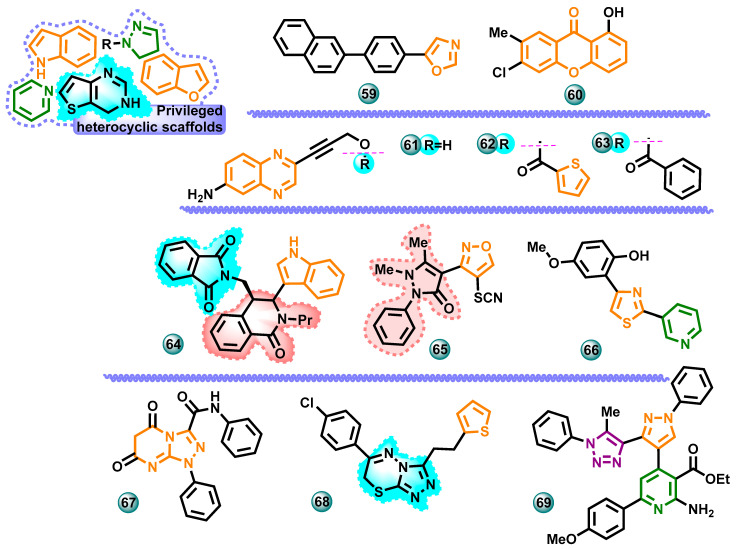
Potential aromatase inhibitors with privileged heterocyclic scaffolds.

New 5-(4-bromophenyl)-1,3-oxazole derivatives were synthesized in 2022 by Golani et al. To assess the aromatase inhibition ability of the synthesized molecules, an automatic docking technique was used. The synthesized compounds were analyzed using spectroscopic methods to determine their structure. In vitro cytotoxicity of six compounds was conducted against MCF-7 cell lines with cisplatin (IC_50_ = 12.46 μM) being used as the reference drug. All tested derivatives showed some activity against estrogen-dependent breast cancer MCF-7 cells. The compound **59** (Figure 13) with the highest activity achieved an IC_50_ of 15.06 µM against MCF-7 [51].

Ramakrishnan et al. synthesized and evaluated the activity of a series of halogenated xanthones. The anticancer properties of all synthesized compounds were evaluated against two cancer cell lines, estrogen-dependent MCF-7 and estrogen-independent MDA-MB-231, using the MTT test. The authors of the study concluded that the newly synthesized molecules are putative aromatase inhibitors. Compound **60** (Figure 13) was the most active compound and exhibited IC_50_ values of 50 μM and 60 μM against the MCF-7 cells and the MDA-MB-231 cells, respectively [52].

New 6-aminoquinoxalinealkynyl derivatives were obtained in 2022. The newly obtained compounds were assessed for their anticancer potential against estrogen-dependent MCF-7 breast cancer cells. For the reference drug ketoconazole, the IC_50_ value was 25.0 μM. Three compounds from the group **61**–**63** (Figure 13) showed moderate cytotoxic activity against MCF-7 with IC_50_ of 69.7, 35.6, and 69.8 μM, respectively [53].

In 2019, Burdzhiev et al. published the results of research on the preparation of *trans*- and *cis*-2-alkyl-3-indolyl-1-oxotetrahydroisoquinoline-4-carboxylic acids. The compounds were assessed for their ability to inhibit aromatase, and the most active compound **64** (Figure 13) from the series reduced the activity of the aromatase enzyme by 40% in vitro at a concentration of 50 μM. Formestane and DL-aminoglutethimide were used as reference drugs. In a concentration of 1 μM, they inhibited the enzyme reaction by up to 20% from the negative control value [54].

The synthesis and assessment of the anticancer activity of a new series of oxazole-antipyrine hybrids were published by Yi X et al. The researchers assessed the influence of new compounds on aromatase activity. They also compared the results with the effects of a referent drug letrozole. The most active compound in this series, compound **65** (Figure 13), exhibited an IC_50_ value of 2.3 nM, which was similar to the activity of letrozole (IC_50_ = 2.8 nM) [55].

The synthesis of pyridine-substituted thiazolylphenol derivatives was described by Ertas et al. Their antiproliferative activity against the MCF-7 and HEK 293 cancer cell lines was then assessed using the MTT assay. The molecule **66** (Figure 13) with the highest anticancer potential showed a reduction in MCF-7 cell survival to 68.32% at a concentration of 10^−4^ M. Moreover, a series of computer tests based on molecular docking were performed to identify possible interactions between the most active structure and the active site [56].

In 2015, Gomha at al. carried out synthetic work, as a result of which they obtained a series of pyrimidine-5,7-(1*H*,6*H*)-dione derivatives by reacting thiobarbituric acid with aromatic amines and formaldehyde and from thiobarbituric acid and hydrazonyl halides, respectively. In order to determine whether the newly synthesized derivatives could inhibit aromatase in vitro, the researchers conducted tests. The results of the tests were favorable when compared to the effects of a reference drug letrozole (IC_50_ = 2.8 nM). The most active compound **67** (Figure 13) in this series showed an IC_50_ value of 1.1 nM. Antitumor activity tests against cell lines were not performed [57].

In the same year, Osmaniye et al. published the results of a study on the synthesis method and biological activity of new heterocyclic derivatives. The compounds were tested for anticancer potential against the MCF-7 cell line by using cisplatin (IC_50_ = 62.08 μM) as a reference. The most active compound **68** (Figure 13) had an IC_50_ value of 3.13 μM. In the in vitro aromatase enzyme inhibition ability test, derivative **68** showed inhibition ability with an IC_50_ of 58 nM (the reference drug was letrozole with IC_50_ = 34 nM). In silico computational tests were also performed for it, in which several parameters derived from the simulation trajectory were verified in terms of the stability of the protein–ligand complex in dynamic conditions [58].

El-Naggar and co-authors synthesized a series of new compounds with anticancer potential using pyrazole-4-carbaldehyde as the starting material. All new molecules were evaluated in vitro for 5α-reductase and aromatase inhibition. In the synthesized series, compound **69** (Figure 13) with the highest activity against aromatase (IC_50_ = 1.42 nM) to the reference drug letrozole (IC_50_ = 2.80 nM) was selected [59].

#### 3.2.4. Aryl/Hetarylhydrazones

**Figure 14:**
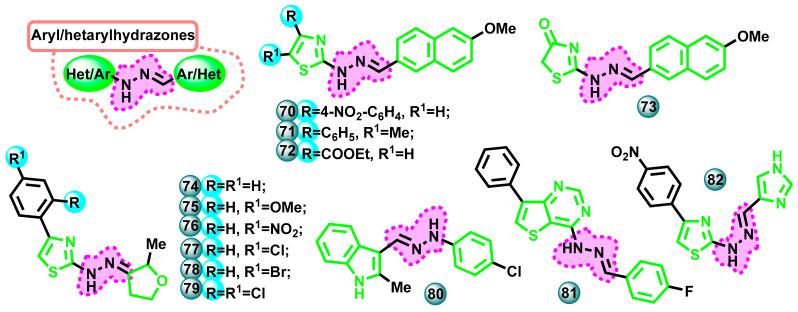
Potential aromatase inhibitors with aryl/hetarylhydrazone moiety in molecules.

A series of new 2-thiazolylhydrazone derivatives using 6-methoxy-2-naphthaldehyde as the starting compound was synthesized by Evren et al. as possible dual monoamine oxidase (MAO) and aromatase inhibitors. The results showed that compound **70** (Figure 14) strongly inhibited MAO-A and MAO-B, while compound **71** (Figure 14) strongly and selectively inhibited MAO-B tests compared to standard drugs. In vitro aromatase inhibition tests showed that compounds **72** and **73** (Figure 14) have high inhibitory potential with IC_50_ values of 31 nM and 42 nM, respectively (the reference drug was letrozole with IC_50_ = 26 nM). Molecular-docking-based tests were also performed for the above-mentioned compounds [60].

Osmaniye et al. conducted research aimed at obtaining new aromatase inhibitors containing thiazole and dihydrofuran ring systems in their structure. The MTT test was performed for the newly synthesized compounds to assess their effect on the cell proliferation of two different lung cell lines (lung cancer cell A549, normal lung fibroblast cell line CCD-19Lu) and an estrogen-dependent breast cancer cell line (MCF-7). In the MTT assay, it was observed that the IC_50_ values of some compounds were higher for the CCD-19Lu cell line than for the A549 and MCF-7 cell lines. Taking into account the results, it was concluded that compounds **74**–**79** (Figure 14) have a favorable safety profile and promising anticancer potential. The apoptotic activity of selected molecules was examined by flow cytometry. The newly synthesized compounds had an apoptotic effect on cancer cell lines. The reference compound used in the tests performed was letrozole. Compound **79** showed the highest activity against the MCF-7 cell line with an IC_50_ value of 8.359 µM. Activity toward aromatase was demonstrated in in silico tests. The most active compounds **77** and **79** had an inhibitory potential of the aromatase enzyme with IC_50_ values of 46 and 30 nM, respectively (IC_50_ value for the reference drug letrozole was 25 nM) [61].

In the same year, Ozcan-Sezer et al. published research results on the aromatase inhibition potential of 2-methyl-indole hydrazones. These compounds were compared with melatonin in two in vitro models, namely a cell-free assay using a fluorescent substrate and an assay against the estrogen-dependent breast cancer cell line MCF-7 in the absence of estrogen and the presence of testosterone. Almost all newly synthesized derivatives at a concentration of 100 µM showed moderate to high aromatase inhibition activity. Indole hydrazones substituted with monochlorine showed stronger inhibitory activity than other tested compounds and were more active than melatonin. Compound **80** (Figure 14) was found to be the most potent aromatase enzyme inhibitor with an IC_50_ value of 8.72 µM (the reference drug was ketoconazole with IC_50_ = 2.39 µM) [62].

New thieno[3,2-*d*]pyrimidine and thienotriazolopyrimidine derivatives were described by Farghaly et al. in 2021. The activity of the compounds was assessed in in vitro tests against the MCF-7 and MDA-MB-231 breast cancer cell lines. Erlotinib and pictilisib were used as reference drugs in the MTT test. Many molecules showed significant antiproliferative activity with IC_50_ values in the range of 0.43–1.31 µM. The highest activity toward MCF-7 (IC_50_ = 0.43 µM) was demonstrated by compound **81** (Figure 14), compared to the reference drug, which was erlotinib (IC_50_ = 5.75 µM) [63].

In 2022, Osmaniye et al. published a paper in which they described the development of a number of new aromatase inhibitors that are also MAO-A and MAO-B inhibitors. The basic idea behind developing this combination of molecular targets for a drug candidate was to prevent the negative effects of aromatase inhibitors on dopaminergic neurons. For this purpose, molecules containing an imidazole ring, which is a structural element designed to ensure the ability to inhibit aromatase, and a thiazolylhydrazone structure with known MAO-B inhibitory activity were developed. Of the obtained derivatives, compound **82** (Figure 14) showed the highest activity against the MCF-7 cell line with an IC_50_ value of 79 nM, compared to doxorubicin with an IC_50_ value of 16.385 µM. The aromatase inhibitory activity of compound **82** had an IC_50_ value of 20 nM. The reference drug used in the study was letrozole (IC_50_ = 24 nM) [64].

#### 3.2.5. Sulfonyl/Amide Containing Molecules and Thioureas as Potential Aromatase Inhibitors

**Figure 15:**
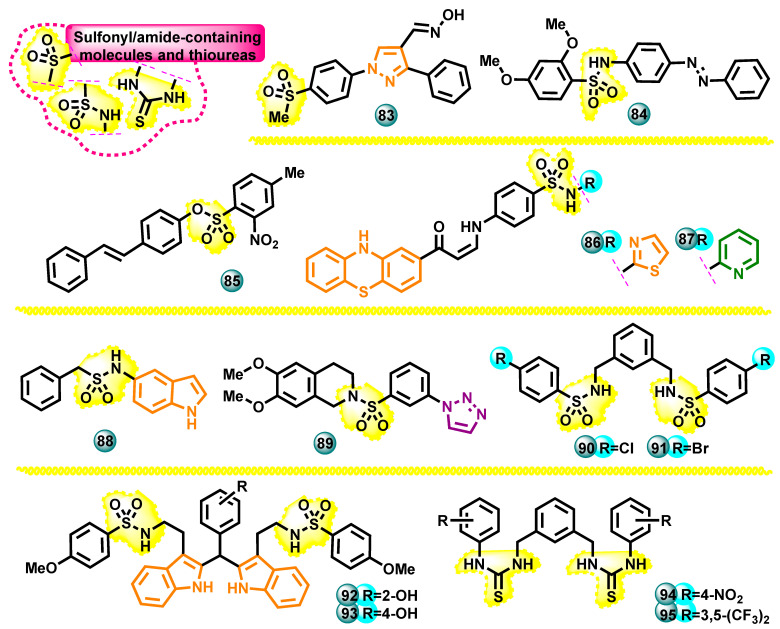
Sulfonyl/amide-containing molecules and thioureas as potential aromatase inhibitors.

Two new series of pyrazole derivatives were synthesized in 2023 by Fadaly et al. In terms of anticancer properties, all synthesized compounds were tested by the National Cancer Institute (NCI) in Bethesda, USA, against 60 human cancer cell lines representing various types of cancer. One of the derivatives containing an internal oxime and sulfon functions in the structure (compound **83**, Figure 15) was the strongest anticancer compound against most of the cell lines used in the tests, in particular breast cancer cell lines MCF-7, human ovarian cancer cell line IGROV1, and human melanoma cell line SK-MEL-5 (IC_50_ = 3.12, 4.28, 4.13 µM, respectively). At the same time, compound **80** turned out to be a strong aromatase inhibitor (IC_50_ = 16.50 µM) compared to the reference drug, which was letrozole (IC_50_ = 15.60 µM) [65].

Several new phenyldiazenylsulfonamide derivatives were developed by Giampietro et al. in 2021. Some of the obtained compounds showed the ability to inhibit the aromatase enzyme in vitro in the micromolar range. Compound **84** (Figure 15) showed an enzymatic inhibition IC_50_ value of 1.6 μM, 50 times better than the IC_50_ of resveratrol (80 μM). These molecules were also assessed for antitumor activity against the human breast cancer cell line MCF-7 using the MTT assay [66].

Research on the synthesis of sulfonate and sulfonamide derivatives of resveratrol as compounds with the potential to inhibit aromatase was described by Fantacuzzi et al. in 2021. Notably, sulfonate derivatives were found to be more active than sulfonamide derivatives. Sulfonate analogs showed good in vitro antiproliferative activity against the MCF-7 breast cancer cell line. The research was carried out using MTT and LDH tests. Compound **85** (Figure 15) with the highest activity had an IC_50_ of 29.2 µM (reference resveratrol IC_50_ = 250 µM) [67].

Ghorab et al. in 2017 conducted research on the design, synthesis, and assessment of the anticancer activity of new phenothiazine derivatives containing a sulfonamide group. Ultimately, twenty new phenothiazine sulfonamide hybrids with promising anticancer activity were obtained. The two most active compounds **86** and **87** (Figure 15) showed cytotoxic activity against the T47D human breast cancer cell line with IC_50_ values of 8.1 and 8.8 µM. The effect of the new molecules in the tests was stronger than that of the reference drug doxorubicin (IC_50_ = 9.8 µM). The molecules were also tested for aromatase inhibition in vitro. The highest inhibitory activity of aromatase was demonstrated by the compound **86** with an IC_50_ value of 5.67 μM (the reference drug was letrozole with IC_50_ = 29.5 μM) [68].

New aromatase inhibitors among arylsulfonamide derivatives containing an indole core were described by Fantacuzzi et al. in 2020. For this purpose, the team synthesized 30 new compounds, for which an aromatase enzyme inhibition test was then performed in vitro*,* using letrozole (IC_50_ = 1.9 nM) as the reference drug. The four synthesized molecules inhibited the protein in the submicromolar range, with an IC_50_ value of 0.16 μM for the most active compound **88** (Figure 15). The cell viability MTT test and cytotoxicity LDH release assay on MCF-7 cells demonstrated a time-dependent and dose-dependent decrease in active metabolizing cells over the time of the culture, starting from a concentration of 100 μM [69].

Thirteen new triazole-tetrahydroisoquinoline derivatives were synthesized by Chamduang et al. and evaluated for their in vitro aromatase inhibition activity. Seven triazoles showed significant inhibitory activity against this enzyme (IC_50_ = 0.07–1.9 µM), compared to the reference drug, which was letrozole (IC_50_ = 1.9 nM). Notably, the most potent aromatase inhibitor **89** (Figure 15) contained a naphthalenyloxymethyl substituent. In cytotoxicity tests against normal cells, this compound did not show significant toxicity [70].

Bis-sulfonamide derivatives that are potential aromatase inhibitors were synthesized by Leechaisit et al. Notably, all but one of the bis-sulfonamide derivatives showed the ability to inhibit the aromatase enzyme in in vitro tests, with IC_50_ results ranging from 0.05 to 11.6 μM. Analogs containing hydrophobic chlorine and bromine groups (compounds **90** and **91**, Figure 15) showed the highest inhibition power of this protein (IC_50_ = 50 and 60 nM). Computer simulations based on molecular docking showed that chloro and bromobenzenesulfonamides may play a role in hydrophobic interactions with Leu477 of aromatase, thus imitating the steroid skeleton of the natural substrate androstenedione [71].

Pingaew et al. synthesized and evaluated in vitro thirty-four indoles containing a sulfonamide moiety. Notably, all indole derivatives inhibited aromatase with IC_50_ values in the range of 0.7–15.3 µM. The highest activity was demonstrated by compounds **92** and **93** (Figure 15) containing a methoxy group, for which aromatase inhibition IC_50_ values were 0.7 and 0.8 µM, respectively (the reference drug was letrozole with IC_50_ = 1.9 nM) [10].

The same authors in 2018 conducted research as a result of which they synthesized three series of thiourea derivatives and then assessed the aromatase inhibition activity of the obtained compounds. These works showed that meta-bisthiourea derivatives are the strongest inhibitors of this enzyme among the newly synthesized molecules, with IC_50_ values of 0.8 µM (compound **94**, Figure 15) and 0.6 µM (compound **95**, Figure 15) (reference letrozole IC _50_ = 1.9 nM). Molecular-docking-based computer simulations showed that thiourea moieties can mimic the steroid skeleton of androstenedione through hydrophobic interactions [72].

#### 3.2.6. Triphenylethylene Derivatives

**Figure 16:**
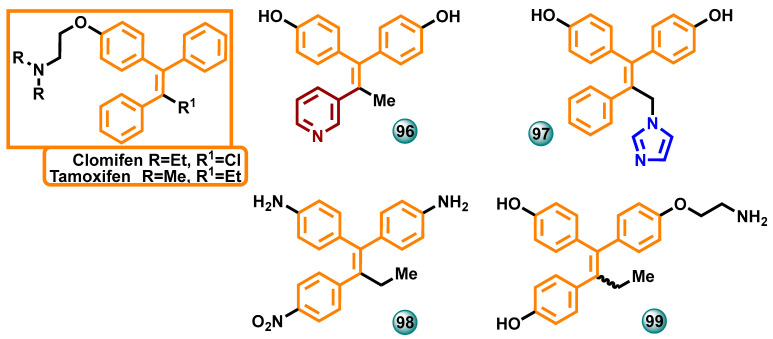
Triphenylethylene derivatives as potential aromatase inhibitors.

In 2021, Caciolla et al. described the results of in silico tests and the synthesis of new tamoxifen analogs and their biological activity in vitro. Activity tests for the obtained compounds were performed against the estrogen-dependent breast cancer cell line MCF-7. The most active compound **96** (Figure 16) showed cytotoxic activity with an IC_50_ of 5.3 μM (the reference drug was letrozole with IC_50_ = 4.1 μM) [73].

Lv W et al. in 2016 synthesized a series of triphenylethylene bisphenol analogs. The compounds were evaluated in vitro for their ability to inhibit aromatase and bind to estrogen receptor α (ER-α) and estrogen receptor β (ER-β) and antagonize the effects of β-estradiol in MCF-7 human breast cancer cells. The most active compound **97** (Figure 16) showed the ability to inhibit aromatase with an IC_50_ of 4.77 nM. The imidazole compounds displayed superior aromatase inhibitory activity compared to the reference drug (*E,Z*)-norendoxifen [74].

The synthesis of a series of compounds with certain structural features of letrozole was described by Zhao et al. in 2016. Ultimately, a group of compounds based on the symmetric diphenylmethylene substructure was obtained. The newly synthesized molecules were tested for activity against aromatase and estrogen receptors in vitro. The most active compound **98** (Figure 16) showed a significant ability to inhibit aromatase (IC_50_ = 62.2 nM), compared to the reference drug, which was (*E*,*Z*)-norendoxifen (IC_50_ = 102.2 nM). At the same time, this compound also showed high activity in attaching to ER-α (EC_50_ = 72.1 nM) and ER-β (EC_50_ = 70.8 nM) receptors [75]. 

The synthesis of new norendoxifene derivatives was described by Lv et al. in 2015. The obtained molecules were tested for activity against human aromatase in vitro. The most active compound in the series, **99** (Figure 16), showed increased aromatase inhibition (IC_50_ = 45.0 nM) capacity compared to norendoxifene (IC_50_ = 102 nM) [76].

## 4. Conclusions 

To summarize, this article presents an overview of work on the synthesis and assessment of the biological activity of new aromatase inhibitors. The main research paths include two directions: the synthesis of derivatives of native ligands, which are compounds with a steroidal structure, and the synthesis of non-steroidal derivatives. In recent years, most research has focused on the biological activity of aromatase inhibition in the group of compounds with a non-steroidal structure and in particular on heterocyclic derivatives. Much less work has been published on compounds with a steroid structure, and most of it did not contain research results on anticancer activity demonstrated against cancer cell lines.

In recent years, many studies have been published indicating the promising activity of azoles, such as triazoles, thiazoles, oxadiazoles, and imidazoles. Some of the tested compounds from this group show promising anticancer activity, better than the reference drugs. The activity of compounds from this group is probably determined by the ability of nitrogen-based heterocyclic systems to form interactions with heme located in the aromatase binding pocket. Based on extensive molecular docking studies, it was found that azole rings have the ability to bind to iron in the center of the heme moiety [77]. Both cytotoxic and cytostatic properties of azole derivatives have been confirmed on various cell lines, with particular emphasis on the hormone-dependent breast cancer cell line MCF-7.

Triazole and imidazole derivatives are characterized by particularly high activity compared to other molecules. The structural element that has a beneficial effect on their activity is the connection to the heterocyclic system via a linker of a phenyl group containing a substituent in the para position. The substituents that clearly positively influence the ability of the molecule to inhibit aromatase are halogen atoms, the nitro group, and the nitrile group. Another structural element that has a positive effect on activity is the connection of the heterocyclic system with the phenyl group using a linker that enables rotation. The results of the collected work also suggest that the presence of the NH group in the linker has a positive effect on the aromatase inhibition ability of the molecule. The use of a sulfone group as a linker in the molecules does not seem to have a beneficial effect on the anticancer activity of the obtained compounds.

Molecules in which a five-membered or six-membered heterocyclic system containing a nitrogen atom is placed in a fused system consisting of a larger number of rings show very high anticancer activity and the ability to inhibit aromatase. In the case of these compounds, the presence of a phenyl ring with a halogen, nitro, or nitrile substituent in their structure has a positive effect on their activity.

The review also includes research on aromatase inhibitors based on a coumarin scaffold. These compounds show some structural similarity to natural flavonoids. These molecules showed high anticancer activity and the ability to inhibit aromatase in tests. For this reason, coumarin derivatives may be an interesting direction of research in the search for new aromatase inhibitors.

Another direction that can be distinguished is the search for aromatase inhibitors among molecules having many phenyl groups. These compounds are structurally similar to the registered aromatase inhibitor drug letrozole. However, in the described molecules, the aromatic system present in letrozole was replaced with a phenolic or pyridine group. The obtained particles did not show any significant activity in the tests. This indicates that the triazole moiety in letrozole is necessary to provide the molecule with aromatase inhibition and anticancer activity.

The most active of all molecules described in recent years were benzimidazole derivatives, for which IC_50_ values against estrogen-dependent breast cancer cells MCF-7 and aromatase were below 20 nM and below 40 nM, respectively.

In conclusion, azole derivatives seem to be the most promising molecules that may become new non-steroidal drugs for the treatment of estrogen-dependent breast cancer.

## Figures and Tables

**Table 1:** The kinase inhibitors in clinical trials, combined with aromatase inhibitors.

Name and Chemical Structure of Kinase Inhibitor	Aromatase Inhibitors Used in Clinical Trials/and Other Drug	ClinicalTrials.gov ID and the Phase of Clinical Trials
***Abemaciclib*** 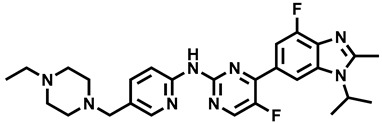	NSAI/FUL ANA, LET ANA ANA	NCT04707196; 4 NCT0224621; 3 NCT02763566; 3 NCT02441946; 2
***Afatinib*** 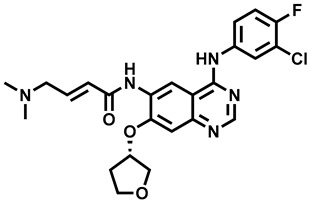	LET	NCT02115048; 2
***Alpelisib*** 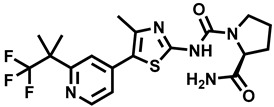	LET	NCT01923168; 3
***Everolimus*** 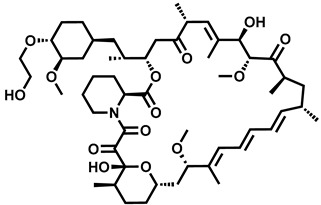	EXE EXE EXE, ANA EXE, ANA EXE LET	NCT01743560; 4 NCT03176238; 3 NCT02137837; 3 NCT01674140; 3 NCT01783444; 2 NCT02520063; 2, 1
***MK-2206*** 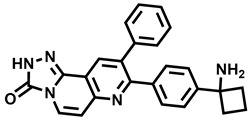	ANA	NCT01776008; 2
***Palbociclib*** 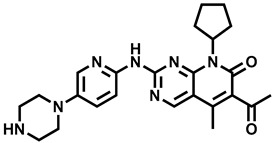	LET LE/FUL AI LET LET ANA LET	NCT02297438; 3 NCT02692755; 3, 2 NCT02040857; 2 NCT02764541; 2 NCT02296801; 2 NCT01723774; 2 NCT02499146; 1
***Ribociclib*** 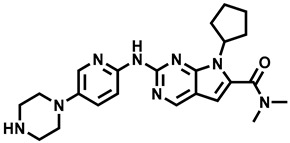	LET LET LET AI EXE	NCT02278120; 3 NCT02941926; 3 NCT03050398; 3 NCT03477396; 2 NCT02732119; 2,1
***Sapanisertib*** 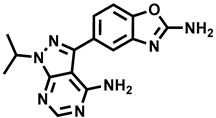	EXE	NCT02049957; 2
***Taselisib*** 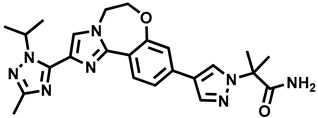	LET	NCT02273973; 2
***Tucatinib*** 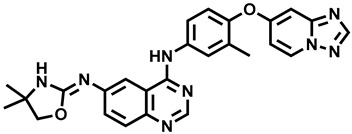	LET	NCT03054363; 2,1

AI—undefined aromatase inhibitor, NSAI—undefined non-steroidal aromatase inhibitor, ANA—anastrozole, EXE—exemestane, FUL—fulvestrant, LET—letrozole.

## Data Availability

Not applicable.
